# A highly efficient tumor-infiltrating MDSC differentiation system for discovery of anti-neoplastic targets, which circumvents the need for tumor establishment in mice

**DOI:** 10.18632/oncotarget.2279

**Published:** 2014-08-04

**Authors:** Therese Liechtenstein, Noemi Perez-Janices, Maria Gato, Fabio Caliendo, Grazyna Kochan, Idoia Blanco-Luquin, Kevin Van der Jeught, Frederick Arce, David Guerrero-Setas, Joaquin Fernandez-Irigoyen, Enrique Santamaria, Karine Breckpot, David Escors

**Affiliations:** ^1^ Division of infection and immunity. Rayne Institute. 5 University Street. WC1E 6JF. London. UK; ^2^ Immunomodulation group. Navarrabiomed-FMS, calle Irunlarrea 3, 31008 Pamplona, Navarra, Spain; ^3^ Cancer Epigenetics group. Navarrabiomed-FMS, calle Irunlarrea 3, 31008 Pamplona, Navarra, Spain; ^4^ Proteomics Unit. Navarrabiomed-FMS, calle Irunlarrea 3, 31008 Pamplona, Navarra, Spain; ^5^ Laboratory of Molecular and Cellular Therapy. Department of Biomedical Sciences. Laarbeeklaan, 103/E, B-1090 Jette. Vrije Universiteit Brussel, Belgium

## Abstract

Myeloid-derived suppressor cells (MDSCs) exhibit potent immunosuppressive activities in cancer. MDSCs infiltrate tumors and strongly inhibit cancer-specific cytotoxic T cells. Their mechanism of differentiation and identification of MDSC-specific therapeutic targets are major areas of interest. We have devised a highly efficient and rapid method to produce very large numbers of melanoma-infiltrating MDSCs *ex vivo* without inducing tumors in mice. These MDSCs were used to study their differentiation, immunosuppressive activities and were compared to non-neoplastic counterparts and conventional dendritic cells using unbiased systems biology approaches. Differentially activated/deactivated pathways caused by cell type differences and by the melanoma tumor environment were identified. MDSCs increased the expression of trafficking receptors to sites of inflammation, endocytosis, changed lipid metabolism, and up-regulated detoxification pathways such as the expression of P450 reductase. These studies uncovered more than 60 potential novel therapeutic targets. As a proof of principle, we demonstrate that P450 reductase is the target of pro-drugs such as Paclitaxel, which depletes MDSCs following chemotherapy in animal models of melanoma and in human patients. Conversely, P450 reductase protects MDSCs against the cytotoxic actions of other chemotherapy drugs such as Irinotecan, which is ineffective for the treatment of melanoma.

Myeloid-derived suppressor cells (MDSCs) have been recognized as major contributors to tumor-induced immunosuppression. Tumor-infiltrating MDSCs strongly inhibit cytotoxic T cells, and their expansion favors tumor progression and metastasis [1, 2]. Counteracting MDSC activities strongly enhances anti-cancer treatments and prolongs survival. Specific MDSC elimination by chemotherapy significantly contributes to anti-tumor efficacy [3-5]. Interestingly, conventional dendritic cells (DCs) remain unaffected by some of these chemotherapy treatments and the mechanisms underlying selective MDSC susceptibility to these drugs are currently unknown. The availability of large numbers of tumor-infiltrating MDSCs would significantly improve research in their biology and functions, and facilitate anti-MDSC drug discovery.

MDSCs in mice comprise a heterogeneous population of immature CD11b^high^ Gr-1^+^ myeloid cells [6]. However, their discrimination from other myeloid cells such as immature DCs, M2 macrophages, monocytes and neutrophils remains somewhat ambiguous. Nevertheless, mouse MDSCs are classified into monocytic (M) and granulocytic (G) subsets, which differ in Ly6C-Ly6G expression profiles. M-MDSCs are Ly6C^high^ Ly6G^−/low^ while G-MDSCs are Ly6C^int/low^ Ly6G^high^. Both subsets suppress immune responses through several pathways, including L-arginine depletion through arginase-1 (arg-1) and inducible nitric oxide synthase (iNOS) activity, increased generation of reactive oxygen species (ROS) and production of immunosuppressive cytokines such as TGF-β [7, 8].

For their study, MDSCs are isolated from the spleen or directly of tumors from a large number of tumor-bearing mice [9-11]. However, spleen MDSCs are phenotypically and functionally different from tumor-infiltrating MDSCs [11, 12]. Moreover, isolated intra-tumor MDSCs are usually contaminated with other myeloid cells, do not proliferate well *ex vivo*, lack plasticity of differentiation and are prone to apoptosis [9, 13, 14]. In addition, low MDSC numbers are obtained from within tumors [12, 15]. *Ex vivo* MDSC production systems have been developed, which rely on incubation of bone marrow (BM) cells with high concentrations of recombinant GM-CSF, alone or in combination with other cytokines, and sometimes supplemented with cancer cell-derived conditioning medium. Nevertheless, these methods achieve MDSC differentiation efficiencies of around 30%-40% of total cells [13, 14]. In practical terms, none of these methods have yet replaced the purification of MDSCs directly from tumors of cancer-bearing mice. Therefore, high-throughput and drug discovery studies with isolated intra-tumor MDSCs are certainly a challenge.

## RESULTS

### *Ex vivo* myelopoiesis within a simulated tumor environment differentiates large numbers of MDSCs

**Figure 1:**
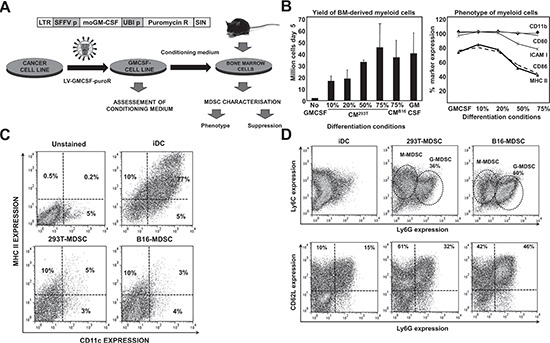
*Ex vivo* myelopoiesis within a simulated tumor environment differentiates bone marrow cells into large numbers of MDSC-like cells **(A)** Experimental scheme for MDSC production. On top, lentivector construct used to express GM-CSF and puromycin resistance gene. Below, schematic representation of the generation of MDSC cells. Cancer cell lines are transduced with the lentivector (LV-GMCSF-PuroR). As a result, transduced cells generate conditioning medium (CM) that simulates the tumor microenvironment. Bone marrow (BM) cells from a single tumor-free mouse are cultured in CM for a minimum of 5 days. **(B)** Left, bar graph representing the number of myeloid cells after a 5 day incubation of BM with the indicated percentages of CM, from 293T or B16F0 cells as indicated. Conventional immature DCs were obtained with recombinant GM-CSF (GM-CSF) following standard protocols. Error bars correspond to standard deviations. Right, percentage of surface expression of the indicated markers, as a function of the increasing percentage of CM^293T^. **(C)** CD11c-MHC II expression profiles are shown as flow cytometry density plots. Percentages of CD11c and MHC II expressing myeloid cells are shown within the graph. Control plots of unstained, immature DCs, 293T- and B16-MDSCs are shown on top of the plots. Myeloid cells were collected on day 8 of differentiation. **(D)** Same as in c but assessing Ly6C-Ly6G (top density plots) and CD62L-Ly6G (bottom density plots) expression profiles in the indicated myeloid cells. LTR, long terminal repeat; SFFV p, spleen focus-forming virus promoter; moGM-CSF, mouse GM-CSF gene; Puromycin R, puromycin resistance gene; UBI p, ubiquitin promoter; SIN, Self-inactivating LTR.

**Figure 2:**
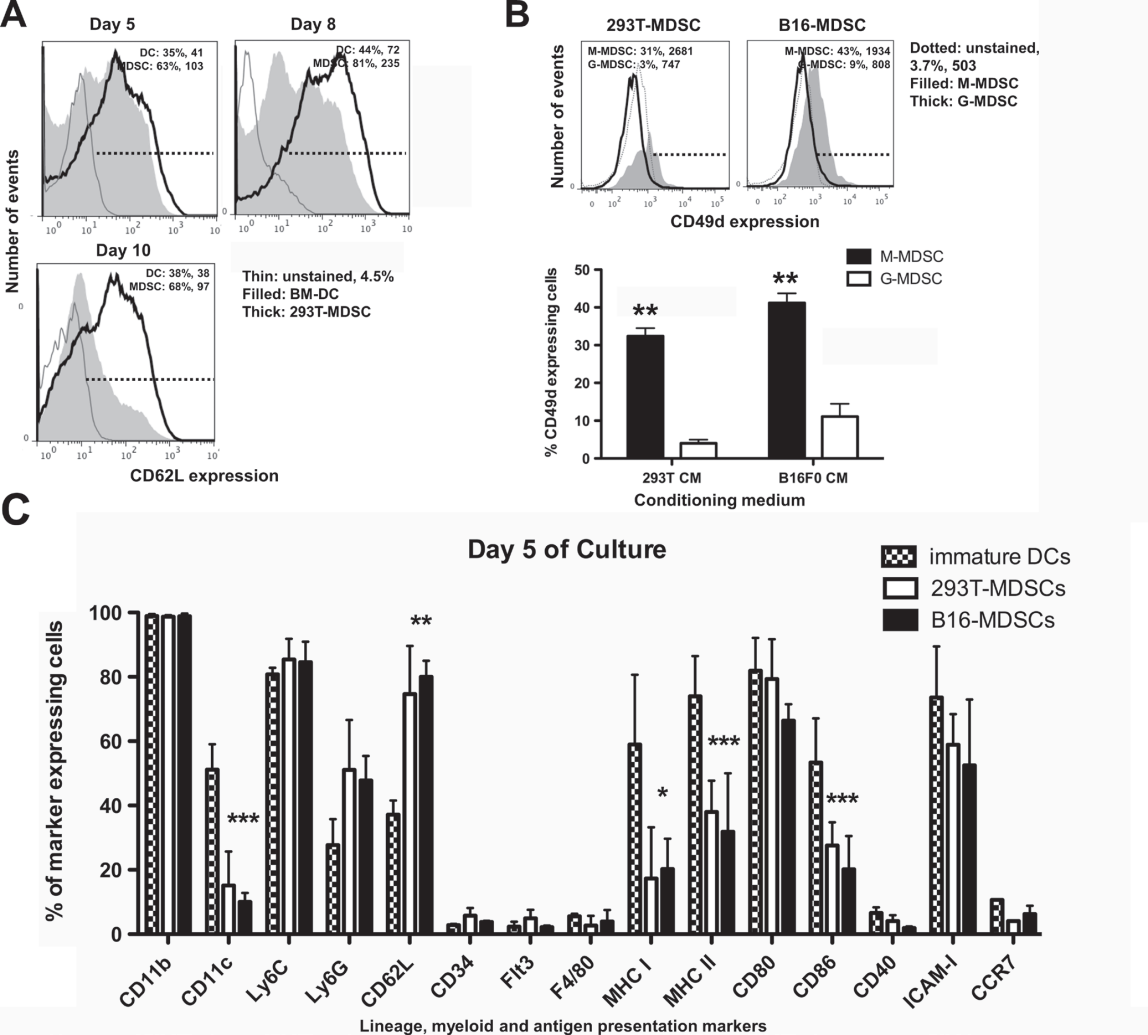
Phenotype profiling of *ex vivo*-differentiated MDSCs **(A)** CD62L expression represented as histograms, in conventional immature DCs and B16-MDSCs at the indicated day of differentiation. Percentages of marker-expressing cells and mean fluorescent intensities (MFI) are shown within the graphs. **(B)** Top histograms represent the expression of CD49d on 293T-MDSCs and B16-MDSCs, for M-MDSCs and G-MDSCs subsets. The column graphs below represent the same data from three independent experiments. Error bars (standard deviations) are shown, and relevant statistical comparisons are indicated within the graph. **(C)** Bar graph representing the percentage of expression of the indicated cell markers in DCs, 293T-MDSCs and B16-MDSCs on day 5 of differentiation, as indicated within the graph. Relevant statistical comparisons are indicated. *, **, ***, represent significant (P<0.05), very significant (P<0.01) and highly significant differences (P<0.001), respectively.

Tumor growth perturbs physiological myelopoiesis in BM leading to the expansion of MDSCs that distribute systemically and infiltrate tumors. To replicate MDSC differentiation without the need of inducing tumors in mice, myelopoiesis within a tumor environment was simulated in cell cultures. To achieve this, B16F0 mouse melanoma cells were genetically modified with a lentiviral vector expressing murine GM-CSF (Fig. 1A). As a control, 293T cells were transduced with the same vector, as this non-neoplastic immortalized cell line is routinely used for protein expression. Culture supernatants of GM-CSF-expressing cells were collected for use as MDSC-conditioning medium (CM^293T^ and CM^B16^). GM-CSF concentration was equivalent as ascertained by ELISA (2.9±0.2 and 2.6±0.1 μg/ml, n=6). Murine BM cells were cultured with CMs at increasing concentrations (Fig. 1A and 1B), leading to strong myeloid differentiation and proliferation. Using 75% of CM routinely yielded myeloid cell numbers between 40 to 70 million cells from BM of a single tumor-free mouse. These yields were comparable to those of standard DC differentiation protocols (Fig. 1B). The increase in proportion of CM correlated with decreasing expression of co-stimulatory marker CD86, major-histocompatibility molecule II (MHCII), and intercellular adhesion molecule 1 (ICAM-I) while maintaining high levels of CD11b and CD80 (Fig. 1B). This phenotype did not correspond to that of immature DCs generated simultaneously with standard protocols. To confirm this, extensive phenotype profiling experiments were carried out. Immature DCs co-expressed MHC II and the conventional DC lineage marker CD11c, a feature of differentiated DCs. In contrast, the expression of these markers in CM-derived myeloid cells was low (Fig. 1C). To test whether these cells were MDSCs, the Ly6C-Ly6G expression profile was studied. These myeloid cells exhibited profiles at day five consistent with monocytic (M) (Ly6C^+^, Ly6G^low/neg^) and granulocytic (G) (Ly6C^+^, Ly6G^high^) MDSCs, clearly distinct from immature conventional DCs (Fig. 1D). We sought out additional markers such as CD62L and CD49d that could discriminate DCs from MDSCs. CD62L (L-selectin) is a homing surface molecule that is selectively expressed in MDSCs within the myeloid lineage [16], and CD49d is present in highly immunosuppressive M-MDSC subsets recently described [17, 18]. CD62L expression was higher in *ex vivo* MDSCs grown in CM after 8 days in culture compared to conventional DCs, with B16-MDSCs showing an increased Ly6G-CD62L co-expression compared to 293T-MDSCs controls (Fig. 1D). We consistently found that CD62L expression was a reliable MDSC surface marker in our *ex vivo* system. While DCs progressively lost CD62L expression, MDSCs increased its expression (Fig. 2A). In addition, the CD49d+ M-MDSC subset was also present in the *ex vivo*-differentiated MDSC cultures (Fig. 2B). Extensive phenotype analyses, which also included lineage markers of conventional DCs, macrophages, monocytes, immature myeloid cells, and hematopoietic precursors, showed that their phenotype corresponded to MDSCs (Fig. 2C).

### *Ex vivo* differentiated M-MDSCs conserve their proliferative capacities and plasticity of differentiation

**Figure 3:**
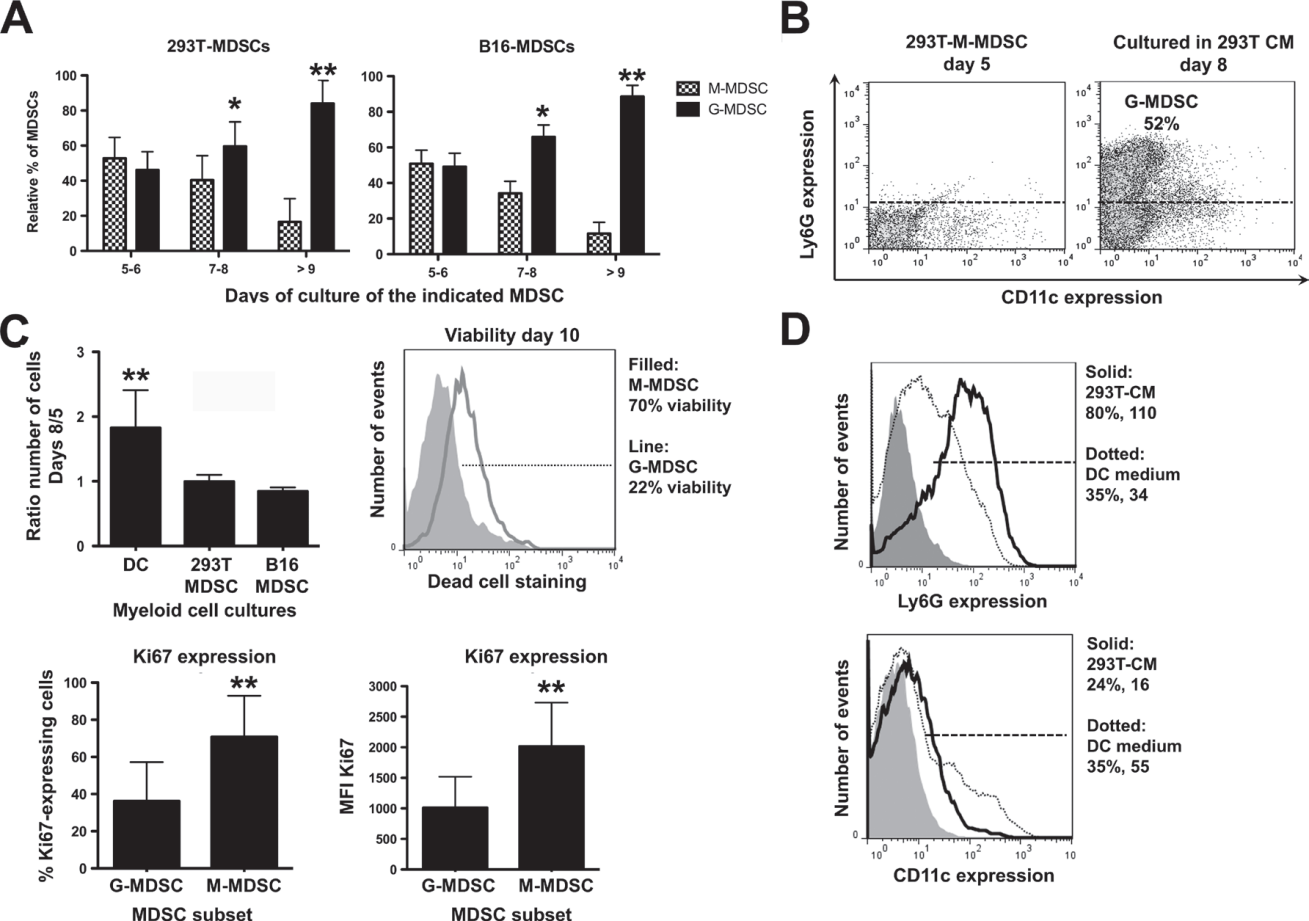
*Ex vivo* monocytic MDSCs are precursors of granulocytic MDSCs, which represent the terminal differentiation stage **(A)** Bar graphs representing the relative proportion of monocytic and granulocytic MDSCs (M-MDSCs, G-MDSCs) in 293T-MDSC and B16-MDSC cultures on the indicated days of differentiation. Relevant statistical comparisons are shown. **(B)** Ly6G-CD11c expression profiles of purified M-MDSCs on day 5 (density flow cytometry plot on the left), and the same cells incubated in CM^293T^ for 3 additional days. The percentage of G-MDSCs is shown within the graph. **(C)** Top left, column graph representing the ratio of the number of cells on days 8 versus 5 in 293T-MDSC and B16-MDSC cultures, to calculate cell growth rate. Top right, dead cell staining with fixable viability stain (FVS) of M-MDSC and G-MDSCs in culture. The proportion of viable cells is shown in the legend. Below left, column graph representing the proportion of Ki67-expressing cells within the G-MDSC and M-MDSC subsets from B16-MDSC cultures, as indicated. Below right, the same but representing Ki67 mean fluorescent intensities (MFI). **(D)** Top histogram, Ly6G expression on day-five 293T-MDSC cultures incubated for three days with either DC medium, or CM^293T^, as indicated within the histogram. Percentages and mean fluorescent intensities are indicated in the legend. The same is represented in the histogram below, but plotting CD11c expression. Relevant statistical comparisons are indicated. *, **, ***, represent significant (P<0.05), very significant (P<0.01) and highly significant differences (P<0.001). Experiments were repeated at least twice.

The relationship between intra-tumor M-MDSCs and G-MDSCs is still uncertain, as G-MDSCs could be either immature recruited neutrophils, or the terminal differentiation stage of M-MDSCs [14, 19]. To test whether M-MDSCs were G-MDSC precursors, their proportion was monitored over time. G-MDSC numbers increased in both 293T-MDSC and B16-MDSC cultures until it comprised the main subset after 10 days (Fig. 3A). In addition, M-MDSCs (CD11b^+^ GR1^+^ Ly6G^neg^) were sorted on day 5 and cultured for 3 days. M-MDSCs converted into G-MDSCs as ascertained by Ly6G up-regulation (Fig. 3B). Interestingly, increase in MDSC numbers stopped from day 5 onwards (Fig. 3C), coinciding with G-MDSC differentiation from M-MDSCs (Fig. 3A). It has been shown that G-MDSCs are terminally differentiated and their viability is compromised *in vivo*. This also held true in our *ex vivo* system. The viability of sorted M-MDSCs and G-MDSCs was assessed by flow cytometry, and G-MDSCs were found to be less viable than M-MDSCs (Fig. 3C).

G-MDSC differentiation is associated with decreased proliferation [14, 19]. To compare the proliferation status of M-MDSCs with that of G-MDSCs in our system, these subsets were sorted and cultured separately overnight. The expression of the proliferation marker Ki67 was significantly higher in M-MDSCs (Fig. 3C). Overall, these results showed that M-MDSCs differentiated into G-MDSCs, and this was accompanied by a loss of proliferation and viability [19]. To definitely prove that the *ex vivo* cultures contained myeloid cells at various differentiation stages, bulk day-five 293T-MDSCs were cultured in DC differentiation medium for 3 additional days. Loss of Ly6G expression and CD11c up-regulation was observed, showing that G-MDSC differentiation could be reverted towards DC differentiation (Fig. 3D). E*x vivo* MDSCs arose from CD11b^neg^ Ly6G^neg^ myeloid cell precursors that conserved a high degree of differentiation potential (not shown).

### *Ex vivo*-differentiated B16-MDSCs resemble *in vivo* melanoma-infiltrating MDSCs

**Figure 4:**
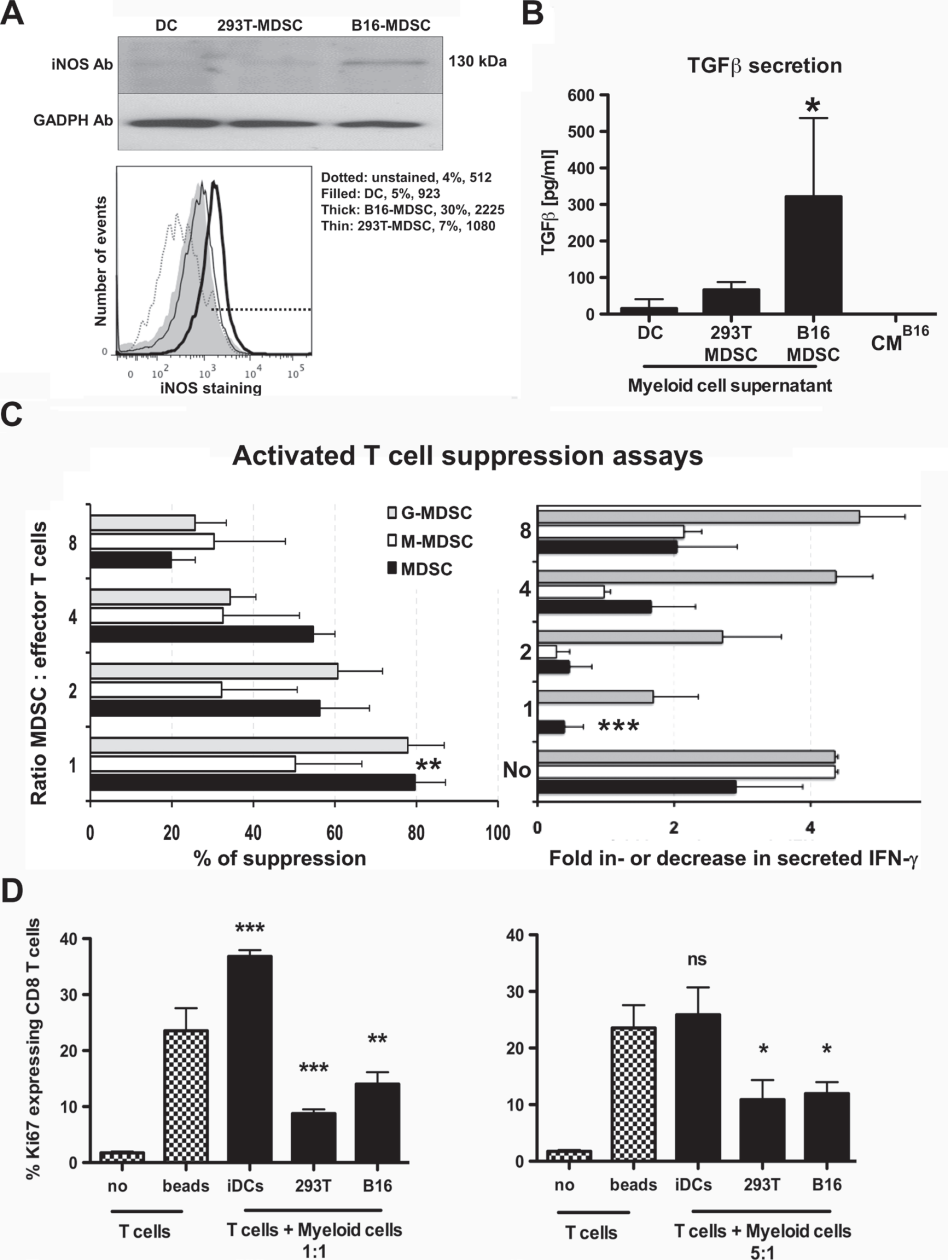
*Ex vivo*-differentiated B16-MDSCs possess characteristics of tumor-infiltrating MDSCs and strongly suppress activated T cells **(A)** Top immunoblot shows iNOS expression in the indicated myeloid cell types. GADPH was detected as a loading control. The histogram below shows the same result but by flow cytometry after intracellular staining with an iNOS-specific antibody. **(B)** The column graph represents secreted bioactive TGF-β in the indicated myeloid cultures, after 7 days of differentiation. 24 hours before TGF-β assessment myeloid cells were plated in medium without CM. Bioactive TGF-β was quantified using a TGF-β-reporter cell line. Standard deviations are represented as error bars. CM^B16^ medium, B16 conditioning medium only. **(C)** The bar graph on the left represents the proliferation inhibition of CD3/CD28-activated CD8 T cells as a titration of MDSC-T cell ratios. Changes in IFN-γ secretion measured during the same experiment are shown in the bar graph on the right. No, represents T cells incubated without MDSCs, three independent experiments. **(D)** Same as c but detecting the expression of the proliferation marker Ki67 using MDSC:T cell ratios as indicated in the figure. Standard deviations are represented as error bars. iDCs, immature DCs. no, no T cell-activatory beads. Relevant statistical comparisons are shown within the graph. *, **, ***, represent significant (P<0.05), very significant (P<0.01) and highly significant (P<0.001) differences, respectively.

In tumor-bearing hosts, spleen and tumor-infiltrating MDSCs differ in phenotype and suppressive activities. Splenic MDSCs are not representative of tumor-infiltrating MDSCs [12]. Interestingly, *ex vivo*-differentiated B16-MDSCs were phenotypically equivalent to *in vivo* B16 melanoma intra-tumor MDSCs on representative markers, which included CD86, MHC II, CD62L, arginase-1 and PD-L1 (Supplementary Fig. S1). To further demonstrate this point, we compared them to 293T-MDSC and immature conventional DC controls. iNOS and TGF-β expression were evaluated as they are signatures of tumor-infiltrating MDSCs. Only B16-MDSCs expressed high levels of iNOS as ascertained by immunoblot and flow cytometry (Fig. 4A). Bioactive TGF-β was quantified with a bioassay [20]. Accordingly, B16-MDSCs produced a higher amount of bioactive TGF-β than either DCs or 293T-MDSCs (Fig. 4B).

Standard T cell suppression assays were performed to confirm their inhibitory activities over anti-CD3/anti-CD28-activated T cells. B16-MDSCs were co-cultured with anti-CD3/CD28-activated CD8 T cells, and their proliferation and IFN-γ production was quantified. Suppression activities of bulk, monocytic and granulocytic populations were assessed. All B16-MDSC subsets inhibited proliferation and IFN-γ production in activated CD8 T cells within a wide range of MDSC:T cell ratios (Fig. 4C). MDSC suppressive activity was further confirmed by assessing Ki67 expression in activated CD8 T cells. In contrast to conventional DCs, MDSCs significantly inhibited Ki67 expression in target T cells (Fig. 4D).

### Tumor-infiltrating MDSCs differ from DCs in key metabolic pathways

To identify MDSC differential intracellular pathways and discover specific therapeutic targets, B16-MDSCs were compared with immature DCs by proteome-scale analyses of relative protein expression levels using iTRAQ isobaric tags coupled to 2D nano-liquid chromatography tandem mass spectrometry. We established stringent cut-off conditions for the identification of differentially-expressed proteins across triplicates from each cell type. 3002 proteins were identified, with a false discovery rate lower than 1%. The expression of 28 proteins was up-regulated in MDSCs while 35 were down-regulated, compared to DCs. Therefore, changes in protein expression between these two cell types under our experimental conditions accounted for 2%. Initially, we had considered the inclusion of purified intra-tumor MDSCs from cancer-bearing mice to compare them to *ex vivo*-differentiated MDSCs by quantitative proteomics. However, the associated cost for obtaining a sample of 10^8^ intra-tumor MDSCs from 600 mice was impractical, in contrast to *ex vivo* MDSC production with a cost of about 80 € for all cell preparations.

**Figure 5:**
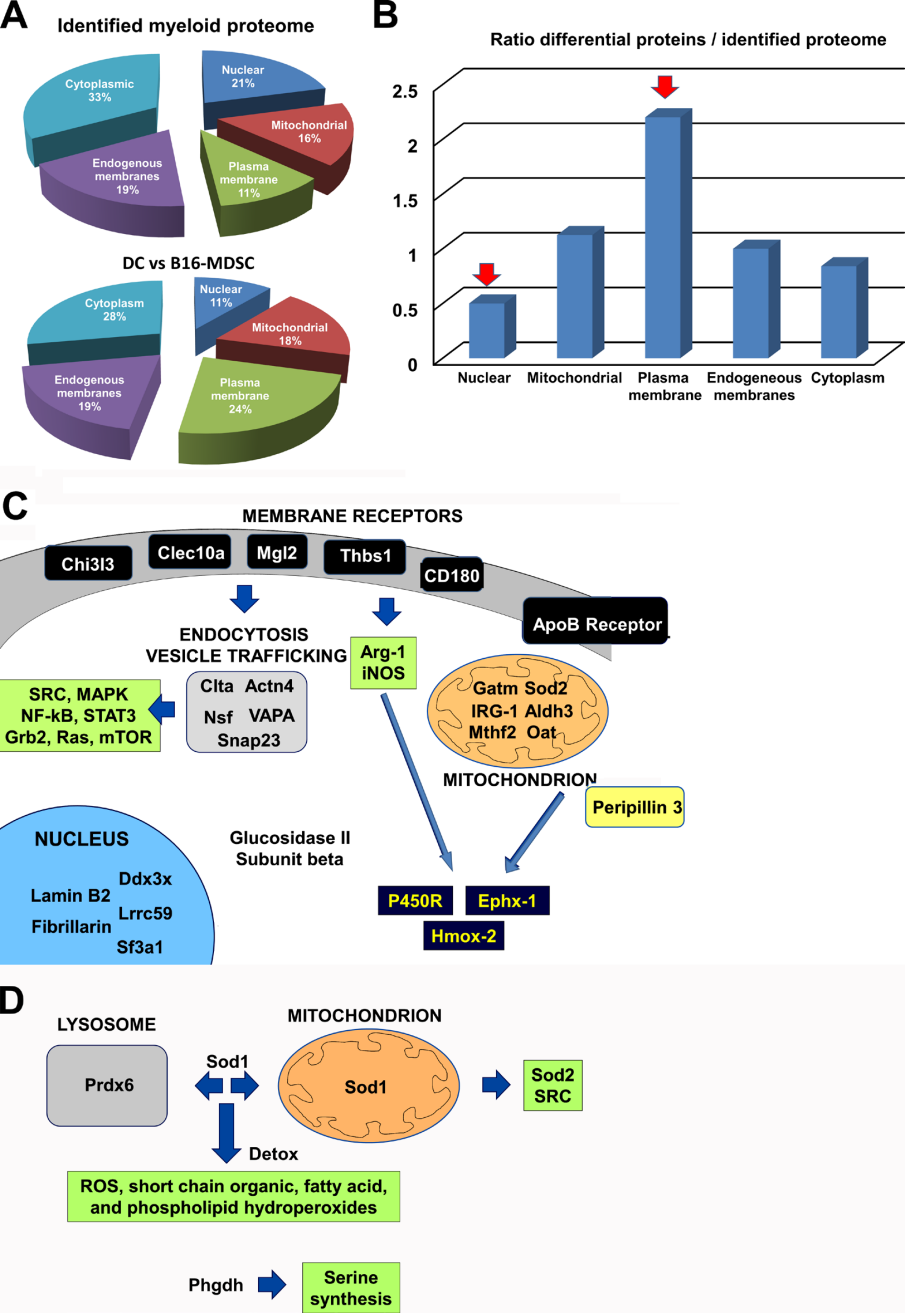
Unbiased comparative quantitative proteomics between *ex vivo* B16-MDSCs and conventional immature DCs **(A)** Top pie chart represents the percentages of total identified proteins, grouped according to the indicated cell locations. Below, pie chart that represents the percentage of differentially-expressed proteins between B16-MDSCs and DCs, grouped according to the indicated cell locations. **(B)** Bar graph that represents the ratio between the percentage of differentially-expressed proteins, related to the total detected proteome, grouped according to cell location. Red arrows indicate protein groups with the highest relative changes in protein expression. **(C)** Schematic diagram integrating the biological relationships and pathways inferred from the up-regulated proteins in B16-MDSCs using String 9.1, DAVID and Panther programs. All significantly increased proteins are indicated, grouped according to cell location. Arrows indicate direct pathways between the indicated protein groups. In dark blue, detoxifying enzymes. Proteins within green boxes indicate pathways which are predicted to be activated from biological interactions of the up-regulated proteins. **(D)** Same as c, but representing differences caused by the tumor environment as highlighted after comparing non-neoplastic 293T-MDSCs with melanoma-specific B16-MDSCs.

Protein identification depended on relative abundance (Fig. 5A). On differentially expressed proteins, the proportion of cellular membrane-associated proteins increased two-fold, while nuclear proteins decreased two-fold (Fig. 5B). To obtain a snapshot on the differentially activated/deactivated intracellular pathways, we undertook a systems biology approach performing molecular network and pathway analyses of differential protein intermediates (Supplementary table 1) using STRING 9.1, DAVID Bioinformatics Resources 6.7 and PANTHER software tools. The results on up-regulated proteins were integrated and summarized in Figure 5C. The expression of some c-type lectin receptors, adhesion molecules and TLR-associated molecules was enhanced in MDSCs (Chi3l3, Clec10a, Mgl2, Thbs1 and CD180). The up-regulation of these molecules was linked to recently found specific targets exploited to deplete MDSCs *in vivo,* such as the S100 proteins [21] (Supplementary Fig. S2). These molecules participate in phagocyte migration to sites of inflammation, NOS and ROS production by Arg-1, NOS1, NOS2 and NOS3, responses to hypoxia and increase in clathrin-dependent endocytosis (Fig. 5C and Supplementary Fig. S2). Accordingly, proteins involved in endocytosis, vesicle trafficking and fusion were clearly increased in MDSCs (Clta, Actn4, Nsf, Snap23, VAPA). All these networks were associated to intracellular signaling pathways known to be active in MDSCs (SRC, Grb2, Ras, Stat3, NF-κB and MAPKs), some of them melanoma MDSC-specific targets such as STAT3 [22] (Fig. 5C and Supplementary Fig. S3).

Increased expression of proteins associated to transcription, splicing and translation was observed, which included RNA helicase Ddx3x, splicing factor 3A (Sf3a1), Lrrc59 (mRNA splicing), lamin B2 (Lmnb2), fibrillarin (Fbl) and glucosidase II subunit beta (Prkcsh) (Fig. 5C). These results indicated that *ex vivo* MDSCs were not quiescent cells. MDSCs function under hypoxic conditions [11], and this was supported by our data. Thus, enzymes linked to glycogen/glucose catabolism and aerobic cellular respiration were down-modulated, such as coenzyme Q10 (ubiquinone), NADH dehydrogenase flavoprotein 2, creatine kinase, glycogen phosphorylase and phosphoglucomutase-1 (Supplementary Table 1). Further, MDSCs increased proteins linked to lipid metabolism, including ApoB receptor (lipoprotein endocytosis), peripilin-3 (Plin3, lipid storage, hydrolysis and metabolism), and mitochondrial proteins associated to lipid metabolism and aminoacid synthesis such as aldehyde dehydrogenase (Aldh3), glycine amidinotransferase (Gatm), ornithine aminotransferase (Oat), cis-aconitase decarboxylase (IRG-1) and methylenetetrahydrofolate dehydrogenase (Mtfhd2) (Fig. 5C). Interestingly, the expression of three enzymes involved in β-oxydation was decreased in B16-MDSCs (peroxysomal acyl-CoA oxidase, trifunctional enzyme subunit β, and carnitine O-palmitoyltransferase). In addition, a range of lysosomal enzymes were down-modulated (Supplementary Table 1).

Lipid metabolism, NOS and ROS production generate highly toxic metabolites. A key characteristic of MDSCs was the up-regulation of detoxifying enzymes and ROS scavenger proteins. These proteins included cytochrome p450 reductase (P450R), epoxide hydrolase 1 (Ephx-1), heme oxygenase 2 (Hmox2) and superoxide dismutase 2 (Sod2). These enzymes participate in a coordinated fashion in ROS production, protection from oxidative damage and in NADP/NADPH-dependent metabolism (Fig. 5C and Supplementary Fig. S3).

To identify differences exclusively caused by the tumor environment, the B16-MDSC proteome was compared to that of their non-neoplastic 293T-MDSC counterparts (Supplementary Table 2). The number of detected differential proteins was reduced to 12 (0.4% of 3100 identified proteins). Three proteins were up-regulated and 9 down-modulated, and pointed to an adaptation to oxidative stress (Fig. 5D). B16-MDSCs up-regulated proteins with antioxidant functions (peroxiredoxin 6 and superoxide dismutase 1), and D-3-phosphoglycerate dehydrogenase, which regulates aminoacid synthesis. B16-MDSCs down-modulated proteins involved in aerobic energy metabolism, such as slc2a6 (hexose transporter) and ADP-riboxyl cyclase (CD38). Interestingly, B16-MDSCs also showed decreased expression of trifunctional enzyme subunit α, a regulator of β-oxidation (Supplementary Table 2).

### Quantitative proteomics uncovers P450 reductase as a MDSC-specific chemotherapy target

**Figure 6:**
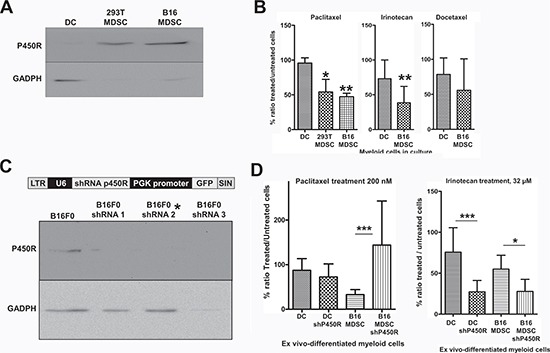
Increased P450R expression renders B16-MDSCs susceptible to Paclitaxel **(A)** Detection of P450R by immunoblot in DCs, 293T-MDSCs and B16-MDSCs as indicated. GADPH expression was used as a loading reference. P450R was very highly expressed in MDSCs compared to DCs (of note, GADPH is expressed at the same levels according to quantitative mass spectrometry). **(B)** Bar graphs representing the ratio (as a percentage) of drug-treated versus untreated DCs, 293T-MDSCs and B16-MDSCs after an overnight treatment with the indicated chemotherapy drugs, at known cytotoxic concentrations for cancer cells. Error bars correspond to standard deviations. **(C)** The lentivector platform used to deliver different P450R-silencing shRNAs is shown above. Below, detection of P450R or GADPH by immunoblot in B16F0 cells transduced with three P450R-silencing lentivectors. The P450R-shRNA2 lentivector was used to modify myeloid cells, indicated with an asterisk. **(D)** Bar graph on the left represents the ratio of paclitaxel-treated versus untreated DCs and B16-MDSCs, unmodified or in which P450R was silenced, as indicated. The bar graph of the right shows the same type of experiments but using Irinotecan to treat cell cultures. LTR, long-terminal repeat; U6, U6 promoter; shRNA, short-hairpin RNA coding sequence; PGK, phosphoglycerolate promoter; SIN, self-inactivating LTR. *, **, ***, indicate significant, very significant and highly significant differences, respectively.

As P450R was highly up-regulated in MDSCs compared to conventional immature DCs, we focused our attention on this protein as a potential MDSC-specific target. To confirm the proteomics data, P450R expression was analyzed by immunoblot in DCs, 293T-MDSCs and B16-MDSCs. P450R was expressed at higher levels in MDSCs than in DCs (Fig. 6A).

P450R participate in activation/metabolism of chemotherapy pro-drugs, which lead to cell growth arrest and cytotoxicity within cancer cells [23, 24]. Moreover, some types of chemotherapy reduce MDSC numbers in treated experimental animals and cancer patients, although the specific mechanism for this selective cytotoxicity remains unclear [3, 4, 24]. We hypothesized that differential P450R expression could be behind it.

Therefore, cells were treated overnight with anti-neoplastic pro-drugs. As we were using melanoma-specific B16-MDSCs, drugs with differing therapeutic efficacies over melanoma were chosen, and for which P450R plays different roles in their mechanisms of action. These included Paclitaxel, Docetaxel and Irinotecan. Paclitaxel is converted to its toxic form by P450/P450R, and it is effective for the treatment of metastasic melanoma by depleting MDSCs [3, 25]; Docetaxel is differently processed by P450 cytochromes and lacks efficacy against melanoma [25-27]; and Irinotecan is activated by carboxylesterases, while neutralized by P450R [28]. Cytotoxic concentrations of these pro-drugs over cancer cells were used on DC and MDSC cultures. The ratio of the number of viable drug-treated *versus* untreated cells was calculated after trypan blue staining. DCs remained largely viable with no significant decrease in cell numbers after Paclitaxel treatment, while the number of treated MDSCs was significantly lower than corresponding untreated controls. Paclitaxel exhibited a higher specificity for MDSCs than Irinotecan (Fig. 6B). Docetaxel treatment affected DCs and MDSCs similarly.

To confirm that P450R caused Paclitaxel-mediated selective inhibitory effects, BM cells were tranduced with a lentivector encoding a P450R-specific shRNA (Fig. 6C), after which DCs and B16-MDSCs were differentiated. P450R silencing conferred protection to Paclitaxel treatment (Fig. 6D), unlike a PD1-specific shRNA used as a control (not shown). As P450R neutralizes Irinotecan, it was used as a control. As expected, P450R silencing both in DCs and B16-MDSCs significantly increased Irinotecan's efficacy. These results confirmed that P450R expression is elevated in MDSCs, and that it confers susceptibility to Paclitaxel, a chemotherapeutic pro-drug that depletes MDSCs *in vivo*.

## DISCUSSION

Isolation, culture and *ex vivo* differentiation of tumor-infiltrating MDSCs are still challenging, time-consuming and expensive. Most current *ex vivo* MDSC differentiation protocols rely on recombinant GM-CSF (usually from bacterial expression systems) as a differentiation factor, although efficiencies do not usually surpass 30% to 40% [13].

We reasoned that endogenous GM-CSF expression by lentivector transduction from melanoma cells would increase the efficiency of MDSC differentiation from BM. In this way, myelopoiesis within a tumor environment was simulated. A large number of MDSCs were obtained, equivalent in phenotype and suppressive functions to those from the same tumor type as used for mimicking the tumor environment *in vitro*. This was not directly caused by high GM-CSF concentration, as the use of equivalent amounts of recombinant GM-CSF was less effective and similar to other published work (not shown). *Ex vivo* differentiated B16-MDSCs expressed iNOS and TGF-β, hallmarks of tumor-infiltrating MDSCs [8, 19, 29, 30]. We also demonstrated that *ex vivo*-differentiated M-MDSCs retained proliferative as well as differentiation plasticity capabilities [13, 19], and served as precursors of G-MDSC and conventional DCs.

Here we characterized the *ex vivo*-differentiated MDSCs using high-throughput quantitative shotgun proteomics to identify global differences caused by cell type (DC vs MDSC), and by the tumor itself (293T- vs B16-MDSC). Cell type differences accounted for 2% of identified proteins, while 0.4% by the tumor environment.

Our data combined with systems biology approaches provided novel potential MDSC-specific therapeutic targets, and confirmed known targets such as STAT3 [22], Sod2 [31] and S100 proteins [21]. MDSCs and DCs differed in the expression of membrane receptors such as lectin-like receptors, TLR and adhesion molecules. Most importantly, they differed in metabolic status. Our data highlighted active MDSCs under hypoxic conditions, indicated by expression of proteins linked to transcription, splicing, mRNA translation, endocytosis and intracellular vesicle trafficking. These processes require high energy levels, but MDSCs down-regulated proteins participating in aerobic ATP production. To compensate, MDSCs enhanced their lipid metabolism, which provides energy and contributes to amino acid synthesis, but also produces a large number of toxic metabolites. Thus, MDSCs responded by expressing high levels of detoxifying enzymes and ROS scavenger proteins such as P450R, heme oxigenase 2, and Sod2 that is elevated in tumor-associated macrophages and tumor cells [31].

Our data uncovered that P450R expression in melanoma-specific MDSCs sensitized them to Paclitaxel treatment while it protected MDSCs against other chemotherapy drugs such as Irinotecan. This is in agreement with its activatory role for Paclitaxel, and its neutralizing role for Irinotecan. Our data was in agreement with the observed inefficacy of Irinotecan for the treatment of melanoma, in contrast to Paclitaxel [32]. Hence, our results correlated with known *in vivo* therapeutic activities. Importantly, this is the first time that P450R up-regulation is demonstrated in tumor-infiltrating MDSCs, which makes them susceptible to Paclitaxel [3, 25].

Our *ex vivo* MDSC differentiation system is a rapid and reproducible method that overcomes the need of inducing tumors in mice. This system allows the identification of therapeutic targets, study MDSC biology and test a large number of treatments in controlled conditions, at a very low cost.

## MATERIALS AND METHODS

### Cells and mice

293T, B16F0 cells and BM-DCs were grown as described [33, 34]. Approval for the animal studies was obtained from the University College London Animal Ethics Committee, the Animal Ethics Committee of the University of Navarra, and from the Government of Navarra. 293T-GM-CSF and B16F0-GM-CSF were generated and MDSCs were obtained from C57BL/6 murine BM cells as described [34]. T cells were isolated and DC-T cell co-cultures were performed as described [20].

### Treatment with chemotherapy agents

MDSCs and DCs (after 5 days in culture) were treated with 200nM, 50nM and 32 μM of Paclitaxel, Docetaxel and Irinotecan, respectively. Cell viability was assessed after 24 hours of incubation, via trypan blue staining. Chemotherapy drugs were obtained from the Pharmacy department of the Hospital de Navarra, Pamplona, Spain.

### Lentivector production and transduction

The pHIV-SIREN [35] was used as a backbone to clone the following P450R shRNA targets: target 1 (CGGAGGCACATCCTAGCCATT), target 2 GCATCTAATGCACCTGGAATT), target 3(CCTGACCTACTGGTTCATCTT). Lentivectors were produced and titrated as described [33].

### Immunoblot

Immunoblots were performed as described, using the iNOS-specific antibodies [33]. Mouse anti-iNOS antibodies were purchased from Cell Signaling, and anti-GADPH from Calbiochem. Polyclonal anti-P450R antibodies were purchased from Abcam. Peroxidase-conjugated anti-mouse and anti-rabbit antibodies were purchased from DAKO.

### Cell staining and flow cytometry

Surface and intracellular staining were performed as described previously using the indicated antibodies [33]. From BioLegend: Alexa fluor 488-Ly6C, PE-CD3, PE-CD86, APC-CD80, APC-I-A/I-F, Biotin-ICAM I, Biotin-H2kb, Alexa fluor 488-IFN-γ, Biotin-PD-L1, PE-Cy7-Ly6G, PE-CD34, PE-CD14, PE-F4/80, PE-Cy7-streptavidin, APC-streptatividin; From eBioscience: PE-CD4, FITC-IA/IE; From Raybiotech: FITC-class I H-2Kb; From BD bioscience pharmigen: v500-CD4, PE-CD8alpha, APC-CD11b, AF647-Ki67; from Southernbiotech: from Invitrogen: APC-CD11c; PE-streptavidin, FITC-streptavidin; from AbD Serotec: PE-CD62L; from R&D Systems: anti-human/mouse PE-Arginase 1. Viability was established by flow cytometry using the Fixable viability dye-eF780 from eBioscience. From Santa Cruz Biotechnology, NOS2-PE. When indicated, cells samples were treated overnight with 100 ng/ml lipololysaccharide (LPS) from Salmonella enterica serotype abortusequi (SIGMA).

### Mass spectrometry-based quantitative proteomics

A global experiment was carried out with three biological replicates in each experimental condition, using B16-MDSC, 293T-MDSC and DC cell pellets.

#### Sample preparation for proteomic analysis

B16-MDSCs and DC cellular pellets were resuspended in lysis buffer containing 7 M urea, 2 M thiourea, 4% (v/v) CHAPS, 50 mM DTT. Homogenates were spinned down at 14,000 x rpm for 1 h at 15ºC. Protein concentration was measured in the supernatants with the Bradford assay kit (Bio-rad).

#### Proteomic analysis using iTRAQ approach

A shotgun comparative proteomic analysis of total cell extracts using iTRAQ (isobaric Tags for Relative and Absolute Quantitation) was performed [36]. Gobal experiments were carried out with three biological replicates in each experimental condition.

#### Peptide labeling

Protein extracts (160 μg) were precipitated with methanol/choloroform, and pellets dissolved in 7M urea, 2 M thiourea, 4% (v/v) CHAPS. Protein quantitation was performed with the Bradford assay kit (Bio-Rad). iTRAQ labeling of each sample was performed according to the manufacturer's protocol (ABSciex). Briefly, a total of 80 μg of protein from each B16 and DC cell sample was reduced with 50 mM tris (2-carboxyethyl) phosphine (TCEP) at 60 °C for 1 h, and cysteine residues were alkylated with 200 mM methylmethanethiosulfonate (MMTS) at room temperature for 15 min. Protein enzymatic cleavage was carried out with trypsin (Promega; 1:20, w/w) at 37 °C for 16 h. Each tryptic digest was labelled according to the manufacturer's instructions with one isobaric amine-reactive tags as follows: Experiment DCs vs B16-MDSCs: Tag_113_, DC-1; Tag_114_, DC-2; Tag_115_, DC-3; Tag_116_, B16-1; Tag_117_, B16-2; Tag_118_, B16-3. Experiment B16-MDSCs vs 293T-MDSCs: Tag_113_, B16-1; Tag_114_, B16-2; Tag_115_, B16-3; Tag_116_, 293T-1; Tag_117_, 293T-2; Tag_118_, 293T-3. After 1h incubation, each set of labelled samples were independently pooled and evaporated until < 40 μl in a vacuum centrifuge.

#### Peptide fractionation

To increase proteome coverage, the peptide pool was injected to an Ettan LC system with a X-Terra RP18 pre-column (2.1 × 20mm) and a high pH stable X-Terra RP18 column (C18; 2.1 mm × 150mm; 3.5μm) (Waters) at a flow rate of 40 μl/min. Peptides were eluted with a mobile phase B of 5–65% linear gradient over 35 min (A, 5 mM ammonium bicarbonate in water at pH 9.8; B, 5 mM ammonium bicarbonate in acetonitrile at pH 9.8). 8 fractions were collected, evaporated under vacuum and reconstituted into 20 μl of 2% acetonitrile, 0.1% formic acid, 98% MilliQ-H_2_0 prior to mass spectrometric analysis.

#### Triple-TOF 5600 Mass Spectrometry Analysis

Peptides mixtures were separated by reverse phase chromatography using an Eksigent nanoLC ultra 2D pump fitted with a 75 μm ID column (Eksigent 0.075 × 150). Samples were first loaded for desalting and concentration into a 0.5 cm length 300 μm ID pre-column packed with the same chemistry as the separating column. Mobile phases were 100% water 0.1% formic acid (FA) (buffer A) and 100% Acetonitrile 0.1% FA (buffer B). Column gradient was developed in a 70 min two step gradient from 2% B to 30% B in 60 min and 30%B to 40% B in 10 min. Column was equilibrated in 95% B for 5 min and 2% B for 15 min. During all process, pre-column was in line with column and flow maintained all along the gradient at 300 nl/min. Eluting peptides from the column were analyzed using an AB Sciex 5600 TripleTOF^™^ system. Information data acquisition was acquired upon a survey scan performed in a mass range from 350 m/z up to 1250 m/z in a scan time of 250 ms. Top 25 peaks were selected for fragmentation. Minimum accumulation time for MS/MS was set to 75 ms giving a total cycle time of 2.1 s. Product ions were scanned in a mass range from 100 m/z up to 1700 m/z and excluded for further fragmentation during 15 s. After MS/MS analysis, data files were processed using ProteinPilot^™^ 4.5 software from AB Sciex which uses the algorithm Paragon^™^ (v.4.0.0.0) [37] for database search and Progroup^™^ for data grouping and searched against Uniprot mouse database. False discovery rate was performed using a non-lineal fitting method and displayed results were those reporting a 1% Global False Discovery Rate (FDR) or better. The mass spectrometry proteomics data have been deposited to the ProteomeXchange Consortium (http://proteomecentral.proteomexchange.org) [38] via the PRIDE partner repository with the data set identifiers PXD001103 and PXD001106.

#### Data Analysis

Relative quantification and protein identification were performed with the ProteinPilot^™^ software (version 4.5; ABSciex) using the Paragon^™^ algorithm as the search engine. Each MS/MS spectrum was searched against a database of murine protein sequences (Uniprot complete mouse proteome). The search parameters allowed for cysteine modification by MMTS and biological modifications programd in the algorithm (i.e. phosphorylations, amidations, semitryptic fragments, etc.). Reporter ion intensities were bias corrected for the overlapping isotope contributions from the iTRAQ tags according to the certificate of analysis provided by the reagent manufacturer (ABsciex). The peptide and protein selection criteria for relative quantitation were performed as follows. Only peptides unique for a given protein were considered for relative quantitation, excluding those common to other isoforms or proteins of the same family. Proteins were identified on the basis of having at least one peptide with an ion score above 99% confidence. Among the identified peptides, some of them were excluded from the quantitative analysis for one of the following reasons: (i) The peaks corresponding to the iTRAQ labels were not detected; (ii) the peptides were identified with low identification confidence (<1.0%); (iii) the sum of the signal-to-noise ratio for all of the peak pairs was <6 for the peptide ratios. The protein sequence coverage (95% conf.) was estimated for specific proteins by the percentage of matching amino acids from the identified peptides having confidence greater than or equal to 95% divided by the total number of amino acids in the sequence. Several quantitative estimates provided for each protein by ProteinPilot were utilized: the fold change ratios of differential expression between labelled protein extracts; the p-value, representing the probability that the observed ratio is different than 1 by chance. A decoy database search strategy was also used to estimate the false discovery rate (FDR), defined as the percentage of decoy proteins identified against the total protein identification. The FDR was calculated by searching the spectra against the decoy database generated from the target database. The results were then exported into Excel for manual data interpretation. Although relative quantification and statistical analysis were provided by the ProteinPilot software, an additional 1.3-fold change cutoff for all iTRAQ ratios (ratio <0.77 or >1.3) was selected to classify proteins as up- or down-regulated. Proteins with iTRAQ ratios below the low range (0.77) were considered to be underexpressed, whereas those above the high range (1.3) were considered to be overexpressed.

### Bioinformatic analysis

The proteomic information was analyzed using bioinformatic tools including DAVID (Database for Annotation, Visualization and Integrated Discovery) Bioinformatics Resources (v6.7) and PANTHER (Protein annotation through evolutionary relationship) (http://www.pantherdb.org/) software tools [39-41]. These programs detect and infer differentially activated/deactivated pathways as a result of cell type differences. The identification of specifically up- or dysregulated regulatory/metabolic networks in MDSCs was analyzed by STRING (Search Tool for the Retrieval of Interacting Genes) software (v.9.1) (http://stringdb.org/) [39].

### *In vitro* Suppression Assays

To evaluate the suppressive activity of MDSCs, we performed *in vitro* suppression assays as described (supplemental experimental procedures and [34]). To that end, CD8^+^ T lymphocytes were isolated from the spleen of a C57Bl/6 mouse (Harlan) using the CD8^+^ T cell Isolation Kit II (Miltenyi Biotec, Germany). Cells have been isolated according to the manufacturer's protocol. These CD8^+^ T lymphocytes were labeled with CFSE. Then, cells were washed and resuspended in 5 mL PBS/0,1% bovine serum albumin (BSA). Five ml of 0,5μM CFSE were added to the cell suspension, incubated at 37°C, 5% CO_2_ for 10 minutes, washed in serum free Optimem (Invitrogen), centrifuged 7 minutes at 1500 rpm and resuspended in 5 ml Optimem. Cells were plated at 10^5^ cells/100 μL/96 well. Subsequently, the cells were either left unstimulated or were stimulated with a 1/800 dilution of anti-CD3/anti-CD28 coated beads (Invitrogen). To obtain pure Ly6G^+^ or Ly6C^+^ MDSCs, MDSCs were sorted using the Myeloid-Derived Suppressor Cell Isolation Kit (MiltenyiBiotec, Germany). Bulk and sorted MDSCs were added to the stimulated T cells at varying ratios. Dilution of CFSE was evaluated 3 days later by flow cytometry as a measure of T cell proliferation. Alternatively, supernatants were collected and screened for IFN-γ content using ELISA (eBioScience). Flow cytometry was performed to determine dilution of CFSE. To that end, T cells were stained with AlexaFluor 647-conjugated antibodies against CD3. Data were collected using the FACSCanto Flow Cytometer (BD Biosciences, US) and have been analyzed with software programs FACSDiva. During the analysis, cells were gated according to their forward and side scatter distribution and to CD3 expression. Alternatively, proliferation was assessed in non-CFSE-labeled anti-CD3/CD28-activated T cells by intracellular AlexaFluor 647-conjugated antibodies against Ki67. Here, cells were gated according to CD11b and CD8a expression.

### Statistical analysis

GraphPad Prism, Salstat and SPSS software packages were used for plotting data and statistical analyses. No data was considered an outlier. ELISPOT data were analyzed as described [20, 33]. Mean fluorescence intensities from surface or intracellular staining were analyzed as described using one-way or two-way ANOVAs [20, 33].

## SUPPLEMENTARY FIGURES AND TABLE


