# Supplementary material for: A highly efficient tumor-infiltrating MDSC differentiation system for discovery of anti-neoplastic targets, which circumvents the need for tumor establishment in mice
Source: Oncotarget. 2014 Aug 4;5(17):7843–57. doi: 10.18632/oncotarget.2279 (PMC4202165; doi:10.18632/oncotarget.2279)
Supplement: Supplementary file 1 [file oncotarget-05-7843-s001.pdf]

## SUPPLEMENTARY FIGURES AND TABLES

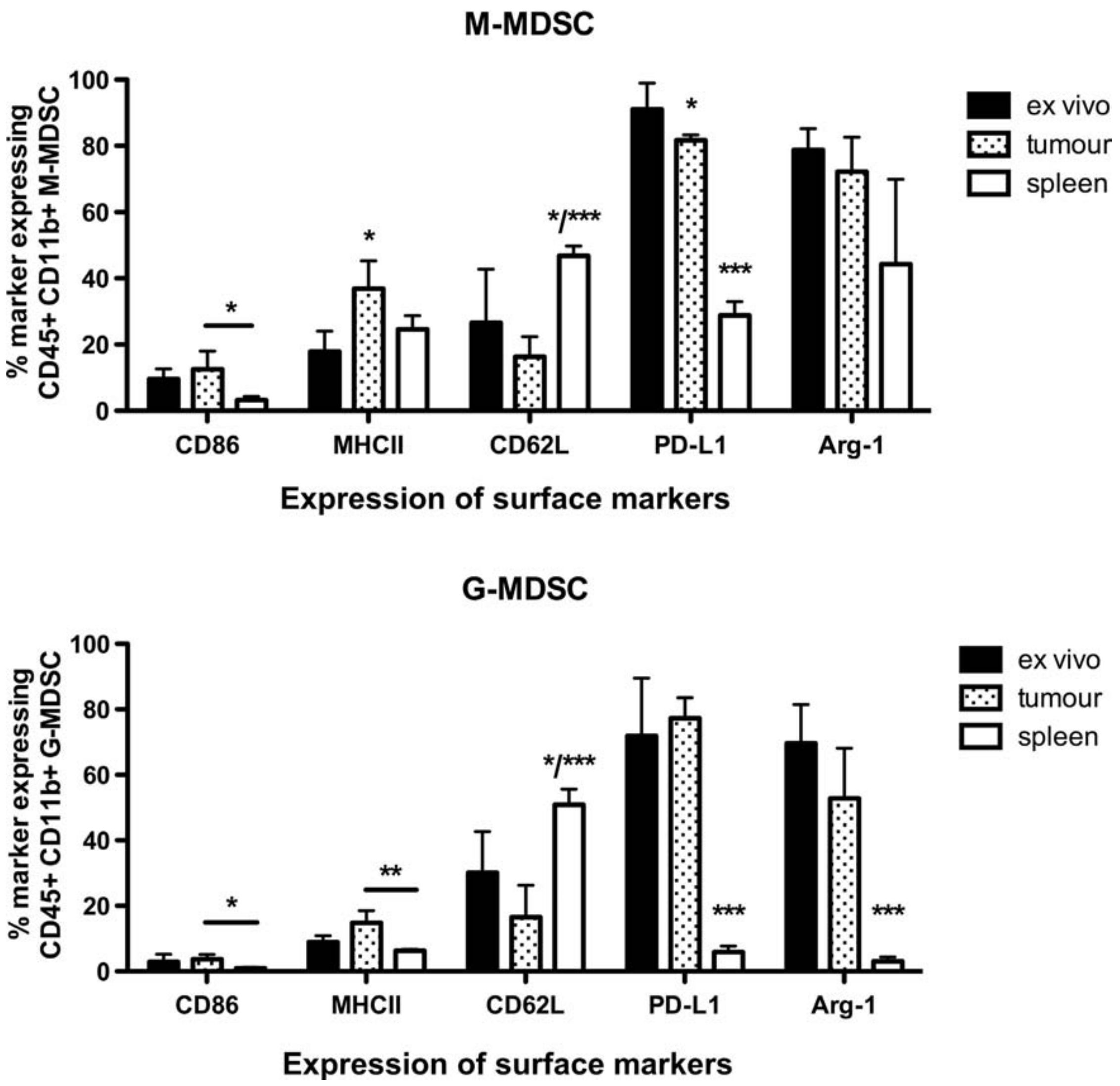


**Supplementary Figure S1: *Ex vivo*-differentiated MDSCs are phenotypically equivalent to their *in vivo* tumor-infiltrating MDSC counterparts but not to splenic MDSCs from tumor-bearing mice.** The graphs represent the percentage of expression of the indicated representative MDSC markers in monocytic (M-MDSC, top graph) and granulocytic (G-MDSC, bottom graph) subsets, in *ex vivo*-differentiated B16-MDSCs compared to splenic and tumor-infiltrating MDSCs from B16 melanoma-bearing mice. Expression of each marker was studied by flow cytometry using specific fluorochrome-conjugated antibodies. *, **, ***, represent significant (P<0.05), very significant (P<0.01) and highly significant (P<0.001) differences, respectively.

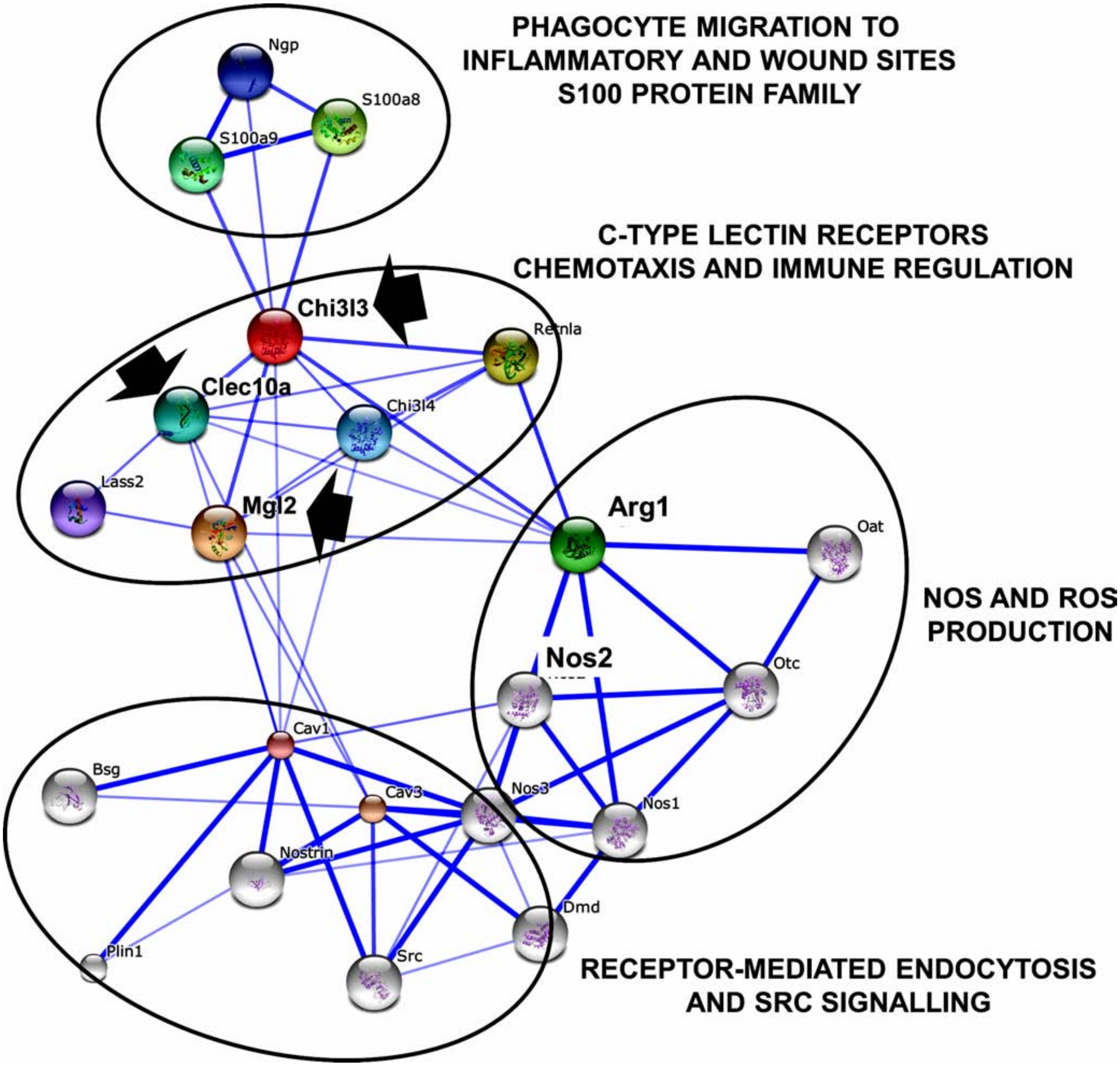


**Supplementary Figure S2: Linkage between up-regulated cell membrane receptors in MDSCs with the S100 protein family, ROS production and Src signaling.** The figure shows a STRING 9.1-derived protein map using the indicated proteins (arrows) as input nodes. These proteins were up-regulated in MDSCs compared to conventional DCs. Functional pathways are grouped and their roles indicated in the figure. S100 family members and Src signaling components have been recently exploited as MDSC-specific therapeutic targets.

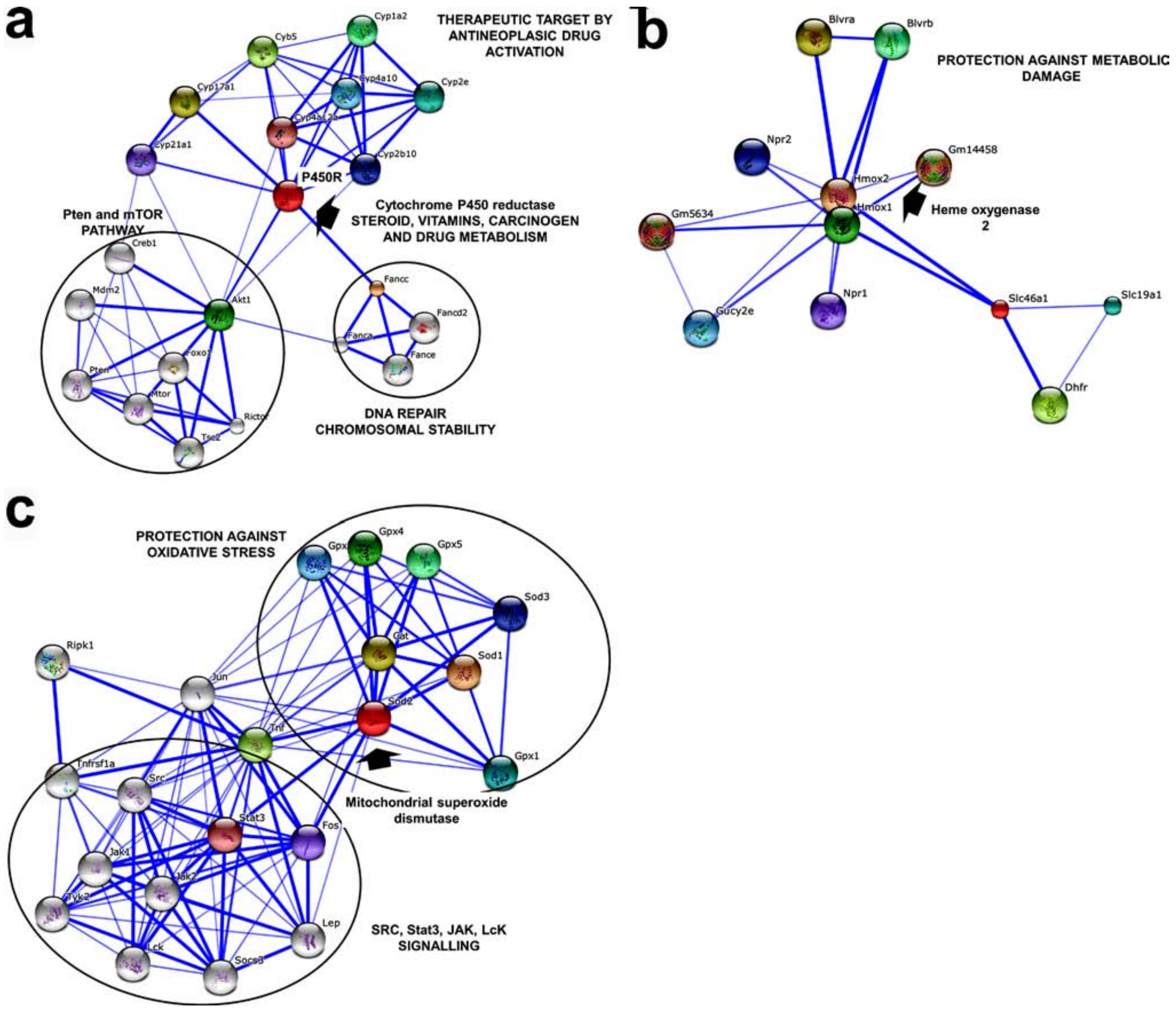


**Supplementary Figure S3: Detoxification, ROS scavengers and protection against oxidative damage in MDSCs.** The figure shows STRING 9.1-derived protein maps using the indicated proteins (arrows) as input nodes. These proteins were up-regulated in MDSCs compared to conventional DCs. Functional pathways are grouped and their roles indicated in the figure. **(a)** P450R, **(b)** heme oxygenase 2 and **(c)** superoxide dismutase were used as input nodes.

**Supplementary Table S1. Differentially expressed proteins between B16-MDSCs and conventional DCs by iTRAQ.** All proteins were identified with >99% confidence (corresponding to a protein score cutoff > 2.0). DC1, DC2, and DC3 together with B16-1, B16-2, and B16-3 correspond to three independent biological replicates from DCs and B16-MDSCs respectively. Ratio corresponds to the protein reporter ion intensity originating from DC2 (Tag114), DC3 (Tag115), B16-1 (Tag116), B16-2 (Tag117), B16-3 (Tag118) relative to DC1 (Tag113), or with 293T-1, 293T-2 and 293T-3 as indicated. Proteins were considered to show a significant downward or upward trend if their expression ratios were < 0.77 or > 1.3 respectively. Ratios indicating differential expression (p-value < 0.05) are in bold. The complete set of proteins identified is provided in Data set 1.

**B16-MDSC vs DC DIFFERENTIALLY EXPRESSED PROTEINS**

| Gene symbol | Access Numb | Protein name | Ratio | | | | | P-value | | | | | %Cov 95% | Unique peptides |
|---|---|---|---|---|---|---|---|---|---|---|---|---|---|---|
| | | | DC2: DC1 | DC3: DC1 | B16-1 DC1 | B16-2 DC1 | B16-3 DC1 | DC2: DC1 | DC3: DC1 | B16-1 DC1 | B16-2 DC1 | B16-3 DC1 | | |
| | | ***Down-regulated proteins in B16-MDSCs*** | | | | | | | | | | | | |
| *Anxa5* | P48036 | Annexin A5 | 0.80 | 0.90 | **0.08** | **0.10** | **0.10** | 0.79 | 0.44 | **0.00** | **0.00** | **0.00** | 88 | 36 |
| *Mfge8* | P21956 | Lactadherin | 1.07 | 0.92 | **0.17** | **0.15** | **0.18** | 0.94 | 0.09 | **0.00** | **0.00** | **0.00** | 40 | 19 |
| *Dpysl2* | O08553 | Dihydropyrimidinase-related protein 2 | 0.95 | 1.07 | **0.24** | **0.39** | **0.25** | 0.48 | 0.72 | **0.01** | **0.03** | **0.00** | 46 | 19 |
| *Atp6v0d2* | Q80SY3 | V-type proton ATPase subunit d 2 | 0.94 | 0.80 | **0.30** | **0.43** | **0.36** | 0.52 | 0.13 | **0.00** | **0.00** | **0.00** | 36.5 | 15 |
| *Anpep* | P97449 | Aminopeptidase N | 0.79 | 0.97 | **0.34** | **0.50** | **0.50** | 0.74 | 0.72 | **0.00** | **0.00** | **0.00** | 37.7 | 45 |
| *Lrpap1* | P55302 | Alpha-2-macroglobulin receptor-associated protein | 1.07 | 1.09 | **0.35** | **0.43** | **0.36** | 0.60 | 0.74 | **0.04** | **0.00** | **0.00** | 55.5 | 35 |
| *Acox3* | Q9EPL9 | Peroxisomal acyl-coenzyme A oxidase 3 | 0.86 | 0.82 | **0.40** | **0.70** | **0.55** | 0.48 | 0.21 | **0.00** | **0.05** | **0.01** | 47.7 | 29 |
| *Emc1* | Q8C7X2 | ER membrane protein complex subunit 1 | 1.09 | 0.99 | **0.40** | **0.63** | **0.47** | 0.91 | 0.89 | **0.01** | **0.04** | **0.03** | 25.7 | 28 |
| *Hnrnpab* | Q99020 | Heterogeneous nuclear ribonucleoprotein A/B | 1.19 | 1.10 | **0.52** | **0.56** | **0.46** | 0.57 | 0.56 | **0.01** | **0.01** | **0.00** | 27.7 | 15 |
| *Alox5* | P48999 | Arachidonate 5-lipoxygenase | 0.99 | 0.87 | **0.54** | **0.53** | **0.59** | 0.46 | 0.10 | **0.00** | **0.00** | **0.00** | 28.2 | 27 |
| *Pigl* | Q9ET01 | Glycogen phosphorylase, liver form | 0.80 | 0.94 | **0.58** | **0.56** | **0.62** | 0.22 | 0.21 | **0.00** | **0.00** | **0.00** | 37.2 | 44 |
| *Gdi2* | Q61598 | Rab GDP dissociation inhibitor beta | 0.89 | 0.93 | **0.59** | **0.48** | **0.70** | 0.64 | 0.45 | **0.01** | **0.00** | **0.04** | 58.2 | 26 |
| *Ndufv1* | D3YUM1 | NADH dehydrogenase [ubiquinone] flavoprotein 1, mitochondrial | 0.86 | 0.94 | **0.61** | **0.57** | **0.62** | 0.25 | 0.80 | **0.02** | **0.02** | **0.01** | 51 | 22 |
| *Cct7* | P80313 | T-complex protein 1 subunit eta | 0.93 | 1.16 | **0.62** | **0.65** | **0.67** | 0.22 | 0.94 | **0.01** | **0.03** | **0.01** | 40.8 | 31 |
| *Vat1* | Q62465 | Synaptic vesicle membrane protein VAT-1 homolog | 0.82 | 0.86 | **0.67** | **0.55** | **0.58** | 0.15 | 0.39 | **0.01** | **0.01** | **0.01** | 63.3 | 24 |
| *Cndp2* | Q9D1A2 | Cytosolic non-specific dipeptidase | 0.78 | 0.96 | **0.70** | **0.68** | **0.69** | 0.18 | 0.66 | **0.05** | **0.04** | **0.03** | 66.3 | 29 |

(*Continued*)

| B16-MDSC vs DC DIFFERENTIALLY EXPRESSED PROTEINS | | | | | | | | | | | | | |
|---|---|---|---|---|---|---|---|---|---|---|---|---|---|
| | | | Ratio | | | | | P-value | | | | | |
| Gene symbol | Access Numb | Protein name | DC2: DC1 | DC3: DC1 | B16-1 DC1 | B16-2 DC1 | B16-3 DC1 | DC2: DC1 | DC3: DC1 | B16-1 DC1 | B16-2 DC1 | B16-3 DC1 | %Cov 95% | Unique peptides |
| | | **Tendency to down-regulation in B16-MDSCs** | | | | | | | | | | | | |
| *Hadhb* | Q99JY0 | Trifunctional enzyme subunit beta, mitochondrial | 1.09 | 0.88 | **0.47** | **0.63** | 0.61 | 0.69 | 0.56 | **0.02** | **0.05** | 0.08 | 50.7 | 28 |
| *Npc1* | O35604 | Niemann-Pick C1 protein | 1.10 | 0.96 | **0.47** | **0.57** | 0.75 | 0.43 | 0.37 | **0.00** | **0.01** | 0.10 | 5.4 | 10 |
| *Copb1* | Q9JIF7 | Coatomer subunit beta | 0.96 | 0.82 | **0.51** | **0.53** | 0.77 | 0.52 | 0.59 | **0.02** | **0.04** | 0.24 | 23.7 | 26 |
| *Hnrnpd* | G5E8G0 | Heterogeneous nuclear ribonucleoprotein D, isoform CRA_b | 1.01 | 1.08 | **0.64** | **0.55** | 0.69 | 0.98 | 0.74 | **0.04** | **0.04** | 0.07 | 47.4 | 12 |
| *Fam129b* | Q8R1F1 | Niban-like protein 1 | 0.85 | 1.04 | **0.37** | 0.60 | **0.60** | 0.42 | 0.99 | **0.01** | 0.06 | **0.04** | 17.4 | 14 |
| *Ndufv2* | Q9D6J6 | NADH dehydrogenase [ubiquinone] flavoprotein 2, mitochondrial | 0.95 | 1.02 | **0.38** | 0.38 | **0.26** | 0.93 | 0.75 | **0.01** | 0.09 | **0.03** | 50.4 | 13 |
| *Hnrnpa1* | Q5EBP8 | Heterogeneous nuclear ribonucleoprotein A1 | 1.06 | 0.96 | **0.38** | 0.39 | **0.34** | 0.41 | 0.83 | **0.02** | 0.28 | **0.03** | 50.6 | 15 |
| *Cpt1a* | P97742 | Carnitine O-palmitoyltransferase 1, liver isoform | 1.05 | 0.86 | **0.52** | 0.60 | **0.59** | 0.82 | 0.45 | **0.02** | 0.12 | **0.03** | 16.7 | 18 |
| *Ptgs1* | P22437 | Prostaglandin G/H synthase 1 | 0.79 | 0.85 | **0.61** | 0.70 | **0.65** | 0.41 | 0.30 | **0.03** | 0.13 | **0.05** | 35.2 | 28 |
| *Bdh1* | Q80XN0 | D-beta-hydroxybutyrate dehydrogenase, mitochondrial | 0.85 | 0.86 | **0.65** | 0.71 | **0.55** | 0.24 | 0.33 | **0.05** | 0.10 | **0.03** | 34.1 | 15 |
| *Atp6v1h* | Q8BVE3 | V-type proton ATPase subunit H | 0.85 | 0.80 | **0.51** | 0.72 | 0.67 | 0.70 | 0.22 | **0.01** | 0.44 | 0.41 | 39.3 | 18 |
| *Lactb* | Q9EP89 | Serine beta-lactamase-like protein LACTB, mitochondrial | 1.14 | 0.84 | **0.55** | 0.70 | 0.67 | 0.67 | 0.59 | **0.03** | 0.18 | 0.16 | 34.1 | 22 |
| *Dock2* | Q8C3J5 | Dedicator of cytokinesis protein 2 | 1.08 | 1.17 | **0.65** | 0.74 | 0.74 | 0.94 | 0.46 | **0.01** | 0.10 | 0.10 | 9.8 | 30 |
| *Gns* | Q8BFR4 | N-acetylglucosamine-6-sulfatase | 0.82 | 0.97 | 0.50 | **0.42** | **0.37** | 0.33 | 0.83 | 0.13 | **0.03** | **0.04** | 18.7 | 11 |
| *Anxa7* | Q07076 | Annexin A7 | 0.83 | 0.96 | 0.61 | **0.63** | **0.64** | 0.38 | 0.49 | 0.06 | **0.01** | **0.01** | 28.3 | 17 |
| *Abhd12* | Q99LR1 | Monoacylglycerol lipase ABHD12 | 1.29 | 0.95 | 0.25 | **0.52** | 0.61 | 0.12 | 0.91 | 0.07 | **0.02** | 0.06 | 45.2 | 19 |
| *Ckb* | Q04447 | Creatine kinase B-type | 1.00 | 1.22 | 0.45 | 0.42 | **0.21** | 0.61 | 0.61 | 0.09 | 0.07 | **0.00** | 37.8 | 14 |
| *Pacsin2* | Q9WVE8 | PKC and casein kinase substrate in neurons protein 2 | 0.96 | 0.96 | 0.46 | 0.67 | **0.36** | 0.64 | 0.88 | 0.14 | 0.17 | **0.01** | 11.7 | 11 |
| *Pgm1* | Q9D0F9 | Phosphoglucomutase-1 | 0.79 | 0.87 | 0.66 | 0.72 | **0.53** | 0.52 | 0.57 | 0.38 | 0.23 | **0.02** | 12.1 | 12 |

| B16-MDSC vs DC DIFFERENTIALLY EXPRESSED PROTEINS | | | | | | | | | | | | | | |
|---|---|---|---|---|---|---|---|---|---|---|---|---|---|---|
| | | | Ratio | | | | | P-value | | | | | | |
| Gene symbol | Access Numb | Protein name | DC2: DC1 | DC3: DC1 | B16-1 DC1 | B16-2 DC1 | B16-3 DC1 | DC2: DC1 | DC3: DC1 | B16-1 DC1 | B16-2 DC1 | B16-3 DC1 | %Cov 95% | Unique peptides |
| | | **Up-regulated proteins in B16-MDSCs** | | | | | | | | | | | | |
| *Clec10a* | F8WHB7 | C-type lectin domain family 10 member A | 1.14 | 1.26 | **1.69** | **1.71** | **1.60** | 0.19 | 0.21 | **0.01** | **0.01** | **0.02** | 51.1 | 9 |
| *Sod2* | P09671 | Superoxide dismutase [Mn], mitochondrial | 0.99 | 0.84 | **1.75** | **1.87** | **2.00** | 0.46 | 0.43 | **0.05** | **0.02** | **0.01** | 58.1 | 16 |
| *Gatm* | Q9D964 | Glycine amidinotransferase, mitochondrial | 0.93 | 1.22 | **1.77** | **2.01** | **1.67** | 0.84 | 0.36 | **0.01** | **0.03** | **0.03** | 49.4 | 24 |
| *Irg1* | P54987 | Cis-aconitate decarboxylase | 1.01 | 1.04 | **1.85** | **2.29** | **2.09** | 0.50 | 0.93 | **0.00** | **0.00** | **0.01** | 39.5 | 15 |
| *Mthfd2* | P18155 | Bifunctional methylenetetrahydrofolate DH/cyclohydrolase | 0.99 | 1.21 | **1.89** | **1.92** | **1.77** | 0.78 | 0.67 | **0.04** | **0.01** | **0.03** | 56 | 16 |
| *Por* | P37040 | NADPH-cytochrome P450 reductase | 0.90 | 0.91 | **1.94** | **2.13** | **2.15** | 0.87 | 0.81 | **0.00** | **0.01** | **0.00** | 46.6 | 34 |
| *Atp1a3* | Q8VCE0 | ATPase, Na+/K+ transporting, alpha 3 polypeptide | 0.89 | 1.14 | **2.07** | **1.85** | **1.79** | 0.58 | 0.84 | **0.00** | **0.01** | **0.05** | 27.1 | 14 |
| *Apobr* | Q8VBT6 | Apolipoprotein B receptor | 1.22 | 1.19 | **2.11** | **2.58** | **2.54** | 0.53 | 0.20 | **0.02** | **0.01** | **0.00** | 31 | 24 |
| *Vapa* | Q9WV55 | Vesicle-associated membrane protein-associated protein A | 1.18 | 0.90 | **2.19** | **2.17** | **2.31** | 0.08 | 0.52 | **0.00** | **0.00** | **0.00** | 67.8 | 14 |
| *Oat* | P29758 | Ornithine aminotransferase, mitochondrial | 0.99 | 1.17 | **2.21** | **2.19** | **2.17** | 0.63 | 0.75 | **0.01** | **0.01** | **0.00** | 65.4 | 34 |
| *Actn4* | P57780 | Alpha-actinin-4 | 0.87 | 0.82 | **2.54** | **2.54** | **2.58** | 0.82 | 0.98 | **0.00** | **0.00** | **0.00** | 68 | 44 |
| *Clta* | B1AWD9 | Clathrin light chain A | 1.06 | 0.97 | **3.28** | **2.15** | **2.83** | 0.87 | 0.99 | **0.01** | **0.04** | **0.03** | 37.1 | 11 |
| *Chi3l3* | O35744 | Chitinase-3-like protein 3 | 0.82 | 0.80 | **3.31** | **3.66** | **3.10** | 0.22 | 0.32 | **0.00** | **0.00** | **0.00** | 67.8 | 34 |
| *Thbs1* | Q80YQ1 | Thrombospondin 1 | 1.13 | 1.07 | **3.44** | **4.41** | **4.02** | 0.88 | 0.59 | **0.00** | **0.00** | **0.00** | 22.1 | 30 |
| *Mgl2* | A9XX86 | MCG21506 | 0.90 | 1.11 | **5.55** | **6.55** | **6.08** | 0.75 | 0.29 | **0.00** | **0.00** | **0.00** | 53.8 | 27 |
| | | ***Tendency to up-regulation in B16-MDSCs*** | | | | | | | | | | | | |
| *Cd180* | Q62192 | CD180 antigen | 1.01 | 1.18 | **1.47** | **1.66** | 1.39 | 0.94 | 0.57 | **0.05** | **0.02** | 0.16 | 22.4 | 12 |
| *Plin3* | Q9DBG5 | Perilipin-3 | 0.88 | 0.86 | **2.27** | **1.80** | 1.56 | 0.58 | 0.65 | **0.01** | **0.02** | 0.12 | 34.1 | 11 |
| *Hmox2* | O70252 | Heme oxygenase 2 | 1.22 | 0.79 | 1.32 | **1.63** | **1.98** | 0.28 | 0.15 | 0.16 | **0.03** | **0.01** | 42.2 | 17 |
| *Fbl* | P35550 | rRNA 2'-O-methyltransferase fibrillarin | 1.21 | 0.95 | 1.33 | **1.79** | **1.64** | 0.60 | 0.83 | 0.37 | **0.03** | **0.04** | 50.7 | 15 |
| *Nsf* | P46460 | Vesicle-fusing ATPase | 0.90 | 0.95 | 1.39 | **1.56** | **1.69** | 0.67 | 0.23 | 0.11 | **0.00** | **0.00** | 46.5 | 34 |
| *Ddx3x* | Q62167 | ATP-dependent RNA helicase DDX3X | 1.21 | 1.14 | 1.39 | **1.67** | **1.60** | 0.91 | 0.55 | 0.07 | **0.02** | **0.04** | 37.7 | 29 |

(*Continued*)

| B16-MDSC vs DC DIFFERENTIALLY EXPRESSED PROTEINS | | | | | | | | | | | | | | |
|---|---|---|---|---|---|---|---|---|---|---|---|---|---|---|
| | | | Ratio | | | | | P-value | | | | | | |
| **Gene symbol** | **Access Numb** | **Protein name** | **DC2: DC1** | **DC3: DC1** | **B16-1 DC1** | **B16-2 DC1** | **B16-3 DC1** | **DC2: DC1** | **DC3: DC1** | **B16-1 DC1** | **B16-2 DC1** | **B16-3 DC1** | **%Cov 95%** | **Unique peptides** |
| *Lrrc59* | Q922Q8 | Leucine-rich repeat-containing protein 59 | 0.96 | 0.81 | 1.79 | **2.78** | **2.70** | 0.35 | 0.10 | 0.35 | **0.00** | **0.00** | 40.7 | 18 |
| *Prkcsh* | O08795 | Glucosidase 2 subunit beta | 0.98 | 1.21 | 2.17 | **2.63** | **2.88** | 0.36 | 0.80 | 0.28 | **0.04** | **0.03** | 38.2 | 23 |
| *Ephx1* | Q9D379 | Epoxide hydrolase 1 | 0.89 | 0.82 | 1.32 | **1.60** | 1.39 | 0.85 | 0.50 | 0.18 | **0.01** | 0.07 | 45.2 | 23 |
| *Snap23* | Q9D3L3 | Synaptosomal-associated protein | 0.93 | 0.96 | 1.43 | **1.91** | 1.79 | 0.64 | 0.59 | 0.10 | **0.02** | 0.06 | 67.8 | 14 |
| *Aldh3b1* | Q80VQ0 | Aldehyde dehydrogenase family 3 member B1 | 1.09 | 0.99 | 1.53 | **2.49** | 2.07 | 0.73 | 0.13 | 0.35 | **0.01** | 0.16 | 45.3 | 21 |
| *Sf3a3* | Q9D554 | Splicing factor 3A subunit 3 | 1.20 | 1.04 | 1.96 | **2.17** | 2.96 | 0.84 | 0.80 | 0.49 | **0.02** | 0.09 | 20.1 | 14 |
| *Lmnb2* | P21619 | Lamin-B2 | 1.19 | 1.22 | 1.77 | 1.39 | **1.74** | 0.81 | 0.61 | 0.06 | 0.18 | **0.01** | 62.4 | 30 |

**Supplementary Table S2.** Differentially expressed proteins between B16-MDSCs and 293T-MDSCs by iTRAQ. All the proteins have been identified with >99% confidence. The table includes gene name, UniprotKB accession number, protein name, ratios and p-values of expression between B16-MDSCs and 293T-MDSCs, percent coverage (95%), and unique peptides. B16-1, B16-2, and B16-3 together with 293T-1, 293T-2, and 293T-3 correspond to three independent biological replicates from B16-MDSCs and 293T-MDSCs respectively. Ratio corresponds to the protein reporter ion intensity originating from B16-2 (Tag114), B16-3 (Tag115), 293T-1 (Tag116), 293T-2 (Tag117), 293T-3 (Tag118) relative to B16-1 (Tag113). Proteins were considered to show a significant downward or upward trend if their expression ratios were < 0.77 or > 1.3 respectively. Ratios deemed to signify differential expression (p-value < 0.05) are in bold. The complete set of proteins identified is provided in Data set 2.

| B16-MDSC vs 293T-MDSC DIFFERENTIALLY EXPRESSED PROTEINS | | | | | | | | | | | | | | |
|---|---|---|---|---|---|---|---|---|---|---|---|---|---|---|
| | | | Ratio | | | | | P-value | | | | | | |
| Gene symbol | Access Numb | Protein name | B16.2: B16.1 | B16.3: B16.1 | 293T.1: B16.1 | 293T.2: B16.1 | 293T.3: B16.1 | B16.2: B16.1 | B16.3: B16.1 | 293T.1: B16.1 | 293T.2: B16.1 | 293T.3: B16.1 | %Cov 95% | Unique Peptides |
| | | ***Up-regulated proteins in B16-MDSCs*** | | | | | | | | | | | | |
| *Phgdh* | Q61753 | D-3-phosphoglycerate dehydrogenase | 0.79 | 0.79 | **0.57** | **0.60** | **0.53** | 0.30 | 0.07 | **0.00** | **0.00** | **0.00** | 18.95 | 10 |
| | | **Tendency to up-regulation in B16-MDSCs** | | | | | | | | | | | | |
| *Prdx6* | Q6GT24 | Peroxiredoxin 6 | 0.81 | 0.83 | **0.76** | **0.76** | **0.72** | 0.24 | 0.17 | 0.17 | **0.02** | 0.07 | 58.48 | 15 |
| *Sod1* | P08228 | Superoxide dismutase [Cu-Zn] | 0.83 | 0.78 | **0.62** | **0.63** | **0.62** | 0.49 | 0.21 | **0.01** | **0.00** | 0.06 | 46.1 | 9 |
| | | **Down-regulated proteins in B16-MDSCs** | | | | | | | | | | | | |
| *Slc2a6* | Q3UDF0 | Protein Slc2a6 | 1.03 | 1.01 | **1.84** | **1.68** | **1.32** | 0.76 | 0.93 | **0.00** | **0.08** | **0.40** | 10.46 | 5 |
| *Cd38* | P56528 | ADP-ribosyl cyclase 1 | 1.05 | 0.90 | **1.81** | **1.58** | **1.65** | 0.53 | 0.25 | **0.01** | **0.01** | **0.04** | 9.539 | 5 |
| *Hadha* | Q8BMS1 | Trifunctional enzyme subunit alpha, mitochondrial | 0.89 | 0.83 | **1.33** | **1.50** | **1.31** | 0.31 | 0.39 | **0.02** | **0.00** | **0.03** | 34.08 | 22 |
| *Vim* | P20152 | Vimentin | 0.83 | 1.00 | **1.72** | **1.69** | **1.40** | 0.06 | 0.99 | **0.00** | **0.00** | **0.02** | 83.26 | 56 |
| *Arg1* | Q61176 | Arginase-1 | 0.85 | 0.88 | **2.47** | **2.52** | **2.63** | 0.18 | 0.71 | **0.01** | **0.00** | **0.00** | 43.96 | 15 |
| | | ***Tendency to down-regulation in B16-MDSCs*** | | | | | | | | | | | | |
| *Sec63* | Q8VHE0 | Translocation protein SEC63 homolog | 1.26 | 1.09 | 1.59 | **1.51** | 1.39 | 0.34 | 0.66 | 0.22 | **0.04** | 0.33 | 2.763 | 5 |

(*Continued*)

| B16-MDSC vs 293T-MDSC DIFFERENTIALLY EXPRESSED PROTEINS | | | | | | | | | | | | | | |
|---|---|---|---|---|---|---|---|---|---|---|---|---|---|---|
| | | | **Ratio** | | | | | **P-value** | | | | | | |
| **Gene symbol** | **Access Numb** | **Protein name** | **B16.2: B16.1** | **B16.3: B16.1** | **293T.1: B16.1** | **293T.2: B16.1** | **293T.3: B16.1** | **B16.2: B16.1** | **B16.3: B16.1** | **293T.1: B16.1** | **293T.2: B16.1** | **293T.3: B16.1** | **%Cov 95%** | **Unique Peptides** |
| *Vti1b* | Q91XH6 | Vesicle transport through interaction with t-SNAREs 1B homolog | 0.98 | 0.00 | **1.67** | **1.37** | 1.31 | 0.69 | 0.91 | **0.03** | **0.01** | 0.22 | 26.29 | 7 |
| *Psen1* | P49769 | Presenilin-1 | 0.99 | , | **1.38** | 1.59 | **1.50** | 0.96 | 0.50 | **0.43** | 0.04 | **0.15** | 8.779 | 3 |
| *Usp25* | P57080 | Ubiquitin carboxyl-terminal hydrolase 25 | 0.99 | 1.05 | **1.59** | 1.35 | 1.46 | 0.94 | 0.70 | **0.03** | 0.08 | 0.14 | 1.232 | 3 |
